# Analysis of Phenolic Acids of Jerusalem Artichoke (*Helianthus tuberosus* L.) Responding to Salt-Stress by Liquid Chromatography/Tandem Mass Spectrometry

**DOI:** 10.1155/2014/568043

**Published:** 2014-08-05

**Authors:** Fujia Chen, Xiaohua Long, Zhaopu Liu, Hongbo Shao, Ling Liu

**Affiliations:** ^1^Key Laboratory of Marine Biology Jiangsu Province, College of Resources and Environmental Sciences, Nanjing Agricultural University, Nanjing 210095, China; ^2^Key Laboratory of Coastal Biology & Bioresources Utilization, Yantai Institute of Coastal Zone Research, Chinese Academy of Sciences (CAS), Yantai 264003, China; ^3^Jiangsu Academy of Agricultural Sciences, Nanjing 210014, China

## Abstract

Plant phenolics can have applications in pharmaceutical and other industries. To identify and quantify the phenolic compounds in *Helianthus tuberosus* leaves, qualitative analysis was performed by a reversed phase high-performance liquid chromatography coupled with tandem mass spectrometry (HPLC-MS/MS) and quantitative analysis by HPLC. Ten chlorogenic acids (CGAs) were identified (3-o-caffeoylquinic acid, two isomers of caffeoylquinic acid, caffeic acid, *p*-coumaroyl-quinic acid, feruloylquinic acid, 3,4-dicaffeoyquinic acid, 3,5-dicaffeoylquinic acid, 1,5-dicaffeoylquinic acid, and 4,5-dicaffeoylquinic acid) by comparing their retention times, UV-Vis absorption spectra, and MS/MS spectra with standards. In addition, four other phenolic compounds, including caffeoyl glucopyranose, isorhamnetin glucoside, kaempferol glucuronide, and kaempferol-3-o-glucoside, were tentatively identified in * Helianthus tuberosus* leaves for the first time. The 3-o-caffeoylquinic acid (7.752 mg/g DW), 4,5-dicaffeoylquinic acid (5.633 mg/g DW), and 3,5-dicaffeoylquinic acid (4.900 mg/g DW) were the major phenolic compounds in leaves of *Helianthus tuberosus* cultivar NanYu in maturity. The variations in phenolic concentrations and proportions in *Helianthus tuberosus* leaves were influenced by genotype and plant growth stage. Cultivar NanYu had the highest concentration of phenolic compounds, in particular 3-o-caffeoylquinic acid and 4,5-dicaffeoylquinic acid compared with the other genotypes (wild accession and QingYu). Considering various growth stages, the concentration of total phenolics in cultivar NanYu was higher at flowering stage (5.270 mg/g DW) than at budding and tuber swelling stages. Cultivar NanYu of *Helianthus tuberosus* is a potential source of natural phenolics that may play an important role in the development of pharmaceuticals.

## 1. Introduction

*Helianthus tuberosus* L. (Jerusalem artichoke), Asteraceae family, is a perennial herb originating from eastern North America. It has been introduced and cultivated widely in the temperate areas for the edible tubers.* H. tuberosus* has tall stem, large leaves, bright yellow flowers resembling those of sunflowers, and fleshy potato-like tubers. As a source of inulin, the tubers have been used as a folk medicine for the treatment of diabetes and rheumatism with a variety of pharmacological activities, such as aperient, cholagogue, diuretic, spermatogenic, stomachic, and tonic [1]. Additionally, the leaves of* H. tuberosus* have been utilized as a folk medicine for the treatment of bone fracture, skin wounds, swelling, and pain [2, 3] with antipyretic, analgesic, anti-inflammatory, and antispasmodic effects [4–6]. Moreover, the stalks and leaves of this plant were also found to possess antioxidant, antimicrobial, antifungal, and anticancer activities [1, 6, 7].

The effective compounds in* H. tuberosus* are coumarins, unsaturatedfatty acids, polyacetylenic derivatives, phenolic compounds, and sesquiterpenes [1]. Recent studies have shown that pharmacological characteristics of* H. tuberosus* were related to its phenolic compounds with antioxidant and radical-scavenging activity; the main phenolic acids in* H. tuberosus* leaves were chlorogenic acids [6]. Chlorogenic acids had inhibitory effects on carcinogenesis in the large intestine, liver, and tongue and protective effects against oxidative stress* in vivo* [8]. More broadly, phenolic acids are widely distributed in plants as the secondary metabolites [9]; some phenolic acids are allelochemicals used to control biological pests [10–12], plant pathogens [13], and weeds [14]. The involvement of phenolics with plant protection and communication makes phenolics pivotal molecules in the responses of plants to their ever-changing environment [15].

Previously, it was demonstrated that the leaves of* H. tuberosus* contained high concentration of phenolic compounds [5]. Phenolics were separated and identified (such as ferulic acids) from the tubers of* H. tuberosus* [16]. However, to date, reports on analysis and identification of phenolic compounds from the leaves of* H. tuberosus* are scarce and only a few phenolics, especially chlorogenic acid and isochlorogenic acids, have been identified and qualitatively analysed [6].

Reversed phase high-performance liquid chromatography coupled to tandem mass spectrometry (HPLC-MS/MS) has been extensively and successfully applied to the online structure elucidation of phenolic compounds in foodstuffs, having advantages of high sensitivity, speed, and low sample consumption [17–24]. In addition, liquid chromatography coupled to tandem mass spectrometry (LC-MS/MS) techniques are useful for elucidating the structures of the active compounds (e.g., nonvolatile phenolic compounds) and distinguishing compounds with identical molecular weights [23, 25].

The objectives of the present work were to identify the phenolic compounds in* H. tuberosus* leaves, using HPLC-MS/MS technique, and to measure the concentration of main phenolics in* H. tuberosus* leaves of different cultivars at different sampling periods from budding stage to maturity (tuber swelling stage) using HPLC.

## 2. Materials and Methods

### 2.1. Chemicals and Materials

Gallic acid was obtained from Sinopharm Chemical Reagent Co., Ltd. (Shanghai, China); and 3-o-caffeoylquinic acid was obtained from Aladdin Reagent Co., Ltd. (Shanghai, China). Other standard samples were obtained from Yuanye Biological Technology Co., Ltd. (Shanghai, China). All other analytical grade chemicals were obtained from Shoude Experimental Equipment Co., Ltd. (Nanjing, China).

The leaves of three* H. tuberosus* cultivars (the wild accession, the southern cultivar NanYu [26], and QingYu originated from northern China) were collected from Dafeng District (Jiangsu, China) in maturity at the end of October 2011. Both cultivars NanYu and QingYu [27, 28] are superior varieties in local areas which have obvious competitive advantages in yield, quality, saline-alkali tolerance, and so on over the wild accession in Dafeng District. The leaves of cultivar NanYu were collected from August to October 2012 at different growth stages including budding, flowering, and tuber swelling stages, respectively.

### 2.2. Extraction of Phenolic Compounds

The air-dried (room temperature) and milled [6] leaves (10 g) of* H. tuberosus* were refluxed under vacuum at 50°C using 70% v/v ethanol (EtOH) for three hours. After evaporation under reduced pressure, the dry residue was redissolved in 25 mL of methanol and used for colorimetric and chromatographic analyses. For HPLC analysis, all samples were filtered through a 0.22 *μ*m cellulose acetate filter (Millipore Corp., Bedford, MA, USA) before injections.

### 2.3. Measurement of Total Phenolic Concentration

The total phenolic concentration (TPC) was determined using the Folin-Ciocalteu reagent with gallic acid as standard [19, 29, 30]. The reaction mixture contained 0.5 mL of test sample, 0.5 mL of the Folin-Ciocalteu reagent freshly prepared in our laboratory, 2.0 mL of 10% w/v sodium carbonate solution, and 3.0 mL of distilled water. After 2 h of reaction under ambient temperature in the dark, the absorbance at 760 nm was measured. A calibration curve with equation: *y* = 0.0029*x* + 0.0107 (*R* ^2^ = 0.9991) was constructed using gallic acid solutions in the range of 1.470–294 mg/L. Results were expressed in milligram gallic acid equivalents per gram of dried sample.

### 2.4. HPLC-MS/MS Analysis

HPLC-MS/MS analysis of phenolics in* H. tuberosus* extracts was performed using an Agilent 1200 series HPLC system (Agilent Technology Co. Ltd., USA), composed of a diode array detector and an Agilent 6400 series triple quadrupole (QQQ) mass spectrometer equipped with an electrospray ionization (ESI) source. Data were collected and processed via a personal computer running Agilent MassHunter workstation (Micromass, Qualitative Analysis Version B.01.03 of Agilent Technology Co. Ltd., USA). A reverse-phase Eclipse XDB-C18 column (250 mm × 4.6 mm, 5 *µ*m, Agilent Technology Co. Ltd., USA) was used for separation. The mobile phases consisted of methanol (A) and 1.0% v/v formic acid aqueous solution (B). Gradient elution was started with 30% of A and ascended to 50% of A in 45 min. The flow rate was kept at 0.8 mL/min, and the column temperature was 30°C. Samples were filtered through a 0.22 *µ*m filter prior to HPLC injection. The injection volume was 5 *µ*L. UV-Vis absorption spectra were recorded online from 200 to 600 nm during HPLC analysis. Phenolics were detected at the wavelength of 330 nm.

Mass and MS/MS spectra were achieved by electrospray ionization (ESI) in negative modes. The voltages used were 4000 V for the source capillary and 10 V for the extraction cone: the source temperature was 150°C and the desolvation temperature was 350°C. The electrospray probe flow was adjusted to 70 mL/min. The ESI-MS and ESI-MS/MS spectra were obtained by scanning from 200 to 1200 *m*/*z*. The MS/MS fragmentations were carried out with 10%–50% energy.

### 2.5. Identification and Quantification of Phenolic Compounds

The phenolic compounds in* H. tuberosus* leaves extracts were identified by comparing their UV-Vis absorption spectra, matching their molecular ions (*m*/*z*) obtained by ESI-MS and ESI-MS/MS chromatographic characteristics with the literature data reported [5, 6] or with available reference standards. The external standard method was used for the quantification of main phenolic acids. Concentrations of 3-o-caffeoylquinic acid, caffeic acid, 3,4-dicaffeoylquinic acid, 3,5-dicaffeoylquinic acid, 1,5-dicaffeoylquinic acid, 4,5-dicaffeoylquinic acid, and so on were calculated with the regression equations from the standard curves. Concentrations were expressed as mg/g dried weight sample (DW).

### 2.6. Statistical Analysis

The TPC and concentration of phenolic compounds in* H. tuberosus* leaves of different cultivars and different growth stages were sources of variation. These data were reported as mean ± SD from triplicate determinations. Statistical analysis was performed with analysis of variance (ANOVA) and statistical significance specified at *P* ≤ 0.05.

## 3. Results

### 3.1. Identification of the Chromatographic Peaks

**Figure 1:**
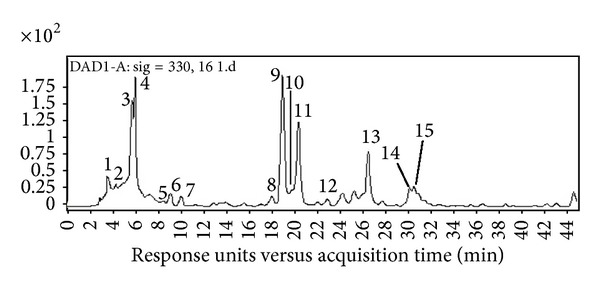
HPLC chromatogram of the ethanol extract of* H. tuberosus* leaves detected at 330 nm. Peak numbers were consistent with those shown in Table 1.

The examination of the chromatograms in a full-scan mode revealed the presence of several compounds, which were positively identified by comparison with available standards.  Figure 1 showed the HPLC chromatogram of ethanol extract from* H. tuberosus* leaves. There were 15 phenolic peaks separated in extracts using the reversed phase C-18 column. As shown in Table 1, peak identification was performed by comparing retention times (*t*_*R*_), UV-Vis spectra, mass, and MS/MS spectra with those of reference standards or literature data.

Classically, chlorogenic acids (CGAs) are a family of esters formed between quinic acid and certain trans-cinnamic acids, most commonly caffeic,* p*-coumaric, and ferulic acids [31]. Fragment ions *m*/*z* 191 and *m*/*z* 179, corresponding to deprotonated quinic acid and caffeic acid fragments, were characteristics of the MS/MS spectra of quinic or caffeic acid derivatives [32].

**Figure 2:**
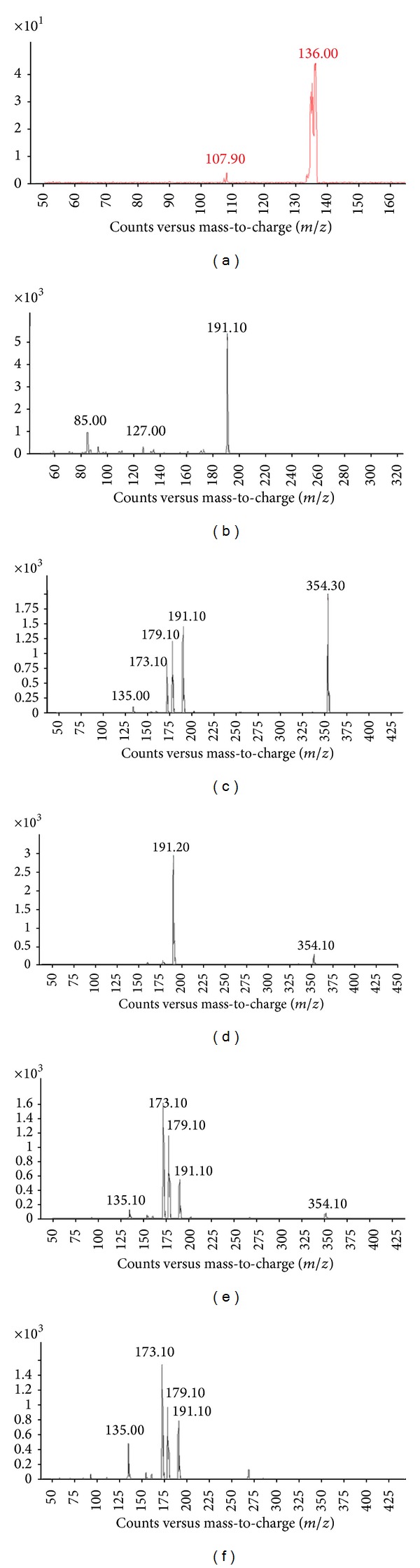
HPLC-MS-MS spectra of phenolic acids (*m*/*z* 179 of caffeic acid (a), *m*/*z* 353 of 3-o-caffeoylquinic acid (b), *m*/*z* 515 of 3,4-dicaffeoylquinic acid (c), *m*/*z* 515 of 3,5-dicaffeoylquinic acid (d), *m*/*z* 515 of 1,5-dicaffeoylquinic acid (e), and *m*/*z* 515 of 4,5-dicaffeoylquinic acid (f)).

Among all the peaks in the chromatogram (Figure 1), peak 9 was quite prominent, indicating a predominant phenolic compound in* H. tuberosus* leaves. This peak presented spectral characteristics of the dicaffeoylquinic acid [5, 6] with UV *λ*_max⁡_ at 242.6 and 327.0 nm and *t*_*R*_ of 19.03 min. The ESI-MS/MS spectra showed [M-H]^−^ at *m*/*z* 515.5, fragment ion [M-C_9_H_6_O_3_]^−^ at *m*/*z* 354.1, and fragment ion [M-H-2C_9_H_6_O_3_]^−^ at *m*/*z* 191.2 (Figures 2(c), 2(d), 2(e), and 2(f)). Compared with the standard, this compound was unambiguously identified as 3,5-dicaffeoylquinic acid. Peaks 8, 11, and 13 had the same spectral characteristics as peak 9 (Table 1), with UV *λ*_max⁡_ at 243.8 and 327.0 nm (peak 8), 243.0 and 329.4 nm (peak 11), and 327.0 nm (peak 13). Based on the MS/MS analyses, the caffeoylquinic acid *m*/*z* 354.1 ion further fragmented to form characteristic *m*/*z* 173.1 [M-H-2C_9_H_6_O_3_-H_2_O]^−^, 135.0 [M-C_7_H_10_O_5_-C_9_H_6_O_3_-COOH]^−^, and 179.0 [M-H-C_9_H_6_O_3_-C_7_H_10_O_5_]^−^ ions [33]. Compared with the standard, these compounds were identified as 3,4-dicaffeoylquinic acid, 1,5-dicaffeoylquinic acid, and 4,5-dicaffeoylquinic acid, respectively [34].

The peaks 2, 3, and 4 were identified as three isomers of caffeoylquinic acids (chlorogenic acid) based on the detailed fragmentation, UV absorption, and also [35]. Previously, the presence of* cis* derivatives of chlorogenic acids was reported in coffee leaves,* Rudbeckia hirta*,* Carlina acaulis*,* H. tuberosus*,* Symphyotrichum novae-angliae*, maté tea (*Ilex paraguariensis*), and leaves of other Asteraceae plants [31, 36–38]. In a negative ion ESI mode, the deprotonated molecule [M-H]^−^ at *m*/*z* 353 and fragment ion [M-H-C_9_H_6_O_3_]^−^ formed from deprotonated quinic acid at *m*/*z* 191 were observed (Figure 2(b)). Fragment ions *m*/*z* 85 and *m*/*z* 93, characteristic of the quinic acid moiety of monoacyl and diacyl chlorogenic acids, defined the parent ions of putative chlorogenic acids [36]. Other fragment ions with different energies such as the caffeic acid unit (*m*/*z* 179.1) and [M-H-C_7_H_12_O_6_]^−^ (*m*/*z* 161.1) were used to distinguish the three isomers [38]. Compared with the standard, peak 3 was identified as 3-o-caffeoylquinic acid.

The peak 5 was pseudomolecular ion [M-H]^−^ at *m*/*z* 179.1 and fragment ions at *m*/*z* 136.0 [M-COO]^−^ and 107.9 [M-COO-CO]^−^ (Figure 2(a)), which were the typical masses of caffeic acid in the negative mode [39]. Fragment ions at *m*/*z* 191 and 179 were also observed in ESI-MS/MS^−^ spectra of peaks 6, 7, and 10 (Table 1) indicating that they were derivatives of quinic acid or caffeic acid. Peak 6 was eluted at 9.19 min (Figure 1), with the molecular ion at *m*/*z* 337.3 [M-H]^−^ and the main fragment ions at *m*/*z* 191.1 [quinic acid-H]^−^ (UV *λ*_max⁡_ at 239.1 nm) and 173.0 [quinic acid-H-H_2_O]^−^ (UV *λ*_max⁡_ at 311.5 nm); this peak was identified as* p*-coumaroyl-quinic acid [31, 36, 40]. Peak 7 (Table 1) was identified as feruloylquinic acid ([M-H]^−^ at *m*/*z* 367 and UV *λ*_max⁡_ at 241.4 and 325.8 nm) [5, 32].

The MS/MS spectrum of peak 10 (Table 1) suggested caffeoyl glucopyranose that possesses both molecular ion *m*/*z* 341.3 and fragmental ion *m*/*z* 179.1 formed by loss of one dehydrated molecule of glucose (Glc) [M-H-(Glc-H_2_O)]^−^, 161.1 [(Glc-H_2_O)-H]^−^ [33, 41]. To our knowledge, caffeoyl glucopyranose has not been previously reported in* H. tuberosus *leaves.

The MS/MS analysis of peaks 12, 14, and 15 (Table 1) showed fragment ions at *m*/*z* 315, 301, and 285, corresponding to methyl quercetin or methoxy kaempferol, quercetin aglycone, and kaempferol, suggesting that they were kaempferol and quercetin glycoside derivatives [33]. Peak 12 had a molecular ion [M-H]^−^ at *m*/*z* 477 and fragment ions at *m*/*z* 315 [M-H-(Glc-H_2_O)]^−^, 300.1 [M-H-(Glc-H_2_O)-CH_3_]^−^, and 270.9 [M-H-(Glc-H_2_O)-CH_3_-CO]^−^, which proved to be isorhamnetin glucoside [23, 33]. Peaks 14 and 15 were possibly kaempferol glucuronide and kaempferol-3-o-glucoside, which have similar fragment ion 285 [kaempferol-H]^−^ and different parent ions 461 [M-H]^−^ and 447 [M-H]^−^ [23, 32, 33, 42–44]. These kaempferol and quercetin glycoside derivatives (peaks 12, 14, and 15) are also the first ever reports in* Helianthus tuberosus* leaves. Their exact structures need further confirmation and additional NMR data will be required.

Phenolics in peak 1 (Table 1) in the HPLC chromatogram were not identified.

### 3.2. Quantification of Phenolics

Concentration of phenolic compounds in* H. tuberosus* leaves of cultivar NanYu was determined by the HPLC method, whereas the concentration of total phenolics was calculated as the sum of the individual phenolic compounds and was also estimated by using the Folin-Ciocalteu method (Table 2). The 3-o-caffeoylquinic acid (7.752 mg/g DW), 4,5-dicaffeoylquinic acid (5.633 mg/g DW), and 3,5-dicaffeoylquinic acid (4.900 mg/g DW) were the major phenolic compounds in* H. tuberosus* leaves, and their concentrations accounted for 33%, 24%, and 21% of the total phenolics, respectively. Among all the quantified phenolics, chlorogenic acids (CGAs) including 3-o-caffeoylquinic acid, caffeoylquinic acids (peaks 2 and 4), caffeic acid, *p*-coumaroyl-quinic acid, feruloylquinic acid, 3,4-dicaffeoylquinic acid, 3,5-dicaffeoylquinic acid, 1,5-dicaffeoylquinic acid, and 4,5-dicaffeoylquinic acid contributed to the total of 22.015 mg/g DW (93% of the total phenolics).

As shown in Table 2, the content of total phenolics calculated as the sum of the individual phenolic compounds was 23.570 mg/g DW, whereas the value obtained by the Folin-Ciocalteu method was 30.159 mg/g DW. The substantial difference between the two values was likely due to the interference of other reducing substances in phenolic extracts, leading to overestimation of total phenolic contents in the Folin-Ciocalteu colorimetric analysis [45, 46].

**Figure 3:**
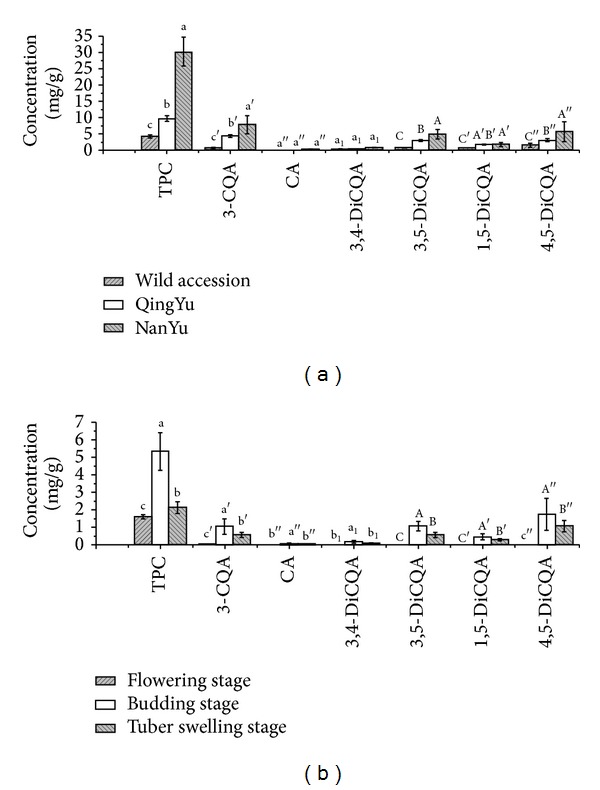
Concentration of phenolics in* H. tuberosus* leaves of different genotypes: (a) in 2011 and growth stages of cultivar NanYu (flowering stage, budding, and tuber swelling stages) and (b) from August to October in 2012. Concentrations were in mg/g dry weight of leaves. Values are expressed as mean ± SD of triplicate measurements; columns with the same letters are not significantly different according to Duncan's test (*P* ≤ 0.05).

Concentration of six main phenolic compounds (peaks 3, 5, 8, 9, 11, and 13 in Table 1) in leaves of different* H. tuberosus* cultivars sampled at different periods from budding to tuber swelling stages was presented in Figure 3. Among the tested genotypes of* H. tuberosus* (Figure 3(a)), NanYu had the highest concentration of phenolic compounds in leaves (around 7-fold higher than the wild accession and 3-fold higher than QingYu).

Caffeic acid was detected in low concentration in all genotypes, whereas concentrations of 3-o-caffeoylquinic acid and 4,5-dicaffeoylquinic acid were considerably higher in all cultivars investigated (Figure 3(a)).

Concentration of total phenolics in leaves of cultivar NanYu was higher at flowering stage (5.270 mg/g DW) than budding and tuber swelling stages (Figure 3(b)), frombudding, flowering to tuber swelling stages.

## 4. Discussion

Phenolic acids are secondary metabolites that are commonly found in plant-derived foods. They have attracted considerable interest due to their many potential health benefits, which are powerful antioxidants and have been reported to demonstrate antibacterial, antiviral, anticarcinogenic, anti-inflammatory, and vasodilatory actions [47]. As allelochemicals, the phenolic acids might play an important role in plant defense against pathogens [48], pests, and weeds [14, 49]. The mechanism of a phenolic with defense, communication, and protection roles was considered as a pivotal molecule in the responses of plants to their ever-changing environment [15].

The variation in concentration of phenolic acids reported here and in the literature was probably due to the isomerisation of chlorogenic acids (CGAs) [50] and different* H. tuberosus* parts considered (tubers, leaves, or whole plants) [6, 9, 31]. In addition, the phenolic profiles of* Helianthus tuberosus* leaves of cultivar NanYu (Dafeng District, Jiangsu, China) were different from previous studies in which the major phenolic compounds were 3-o-caffeoylquinic acid and 1,5-dicaffeoylquinic acid [6] in* H. tuberosus* leaves from Yulin District (Shannxi, China), probably due to different cultivars of* H. tuberosus*, different sampling periods, or different origins.

However, this was to be expected as there were so many environmental factors such as pedoclimatic (soil type, sun exposure, and rainfall) and agronomic factors (growth in greenhouses or fields, biological culture, hydroponic culture, fruit yield per tree, etc.) that could affect phenolics concentration in plants [51]. A degree of ripeness also considerably affected the concentrations and proportions of various phenolics [52]. Thus, cultivar NaYu can be a potential source of natural phenolics, which could have multiple functions (e.g., pharmaceuticals) and could play an important role in plant interactions and ecosystem patterning [14].

## 5. Conclusions

Reversed phase high-performance liquid chromatography coupled with tandem mass spectrometry (HPLC-MS/MS) was successfully employed in the qualitative analysis of phenolic compounds in* H. tuberosus* leaves. Ten chlorogenic acids (CGAs) were identified (3-o-caffeoylquinic acid, two isomers of caffeoylquinic acids, caffeic acid,* p*-coumaroyl-quinic acid, feruloylquinic acid, 3,4-dicaffeoylquinic acid, 3,5-dicaffeoylquinic acid, 1,5-dicaffeoylquinic acid, and 4,5-dicaffeoylquinic acid), and four others (caffeoyl glucopyranose, isorhamnetin glucoside, methoxy kaempferol glucoside, and kaempferol-3-o-glucoside) were tentatively identified for the first time. Quantitative analysis of phenolics indicated that 3-o-caffeoylquinic acid, 4,5-dicaffeoylquinic acid, and 3,5-dicaffeoylquinic acid were the three major phenolic compounds in* H. tuberosus* leaves. The variation in phenolic concentrations and proportions in* H. tuberosus* leaves was characterised in different genotypes and at different sampling periods from budding to tuber swelling stages.* H. tuberosus* cultivar NaYu had the highest concentration of total phenolics and might be a potential source of natural phenolics, which could play an important role in the development of pharmaceuticals.

## Figures and Tables

**Table 1:** Identification of phenolic compounds in *H. tuberosus* leaves by HPLC-MS/MS.

Peaks number	*t*_*R*_ (min)	UV *λ*_max⁡_ (nm)	MW	MS^−^	MS/MS	Identification
1	3.58	246.0, 263.0	360	359.4	297.3, 281.6, 230.9, 135.2	Unknown
2	4.36	214.3, 323.4	354	353.4	191.1, 179.1, 161.1, 135.1, 85.1	Caffeoylquinic acid (isomer of chlorogenic acid)
3	5.78	214.4, 327.0	354	353.4	191.1, 127.0, 85.0	3-o-Caffeoylquinic acid (3-CQA)
4	6.07	237.9, 324.4	354	353.4	191.2, 127, 93.1, 85.0	Caffeoylquinic acid
5	8.57	323.0	180	179.1	136.0, 107.9	Caffeic acid (CA)
6	9.19	239.1, 311.5	338	337.3	191.1, 173.0, 93.0	*p*-Coumaroyl-quinic acid
7	10.12	241.4, 325.8	368	367.3	191.0, 173.1, 134.0, 93.0	Feruloylquinic acid
8	18.09	243.8, 327.0	516	515.5	354.3, 191.1, 173.1, 179.1, 135.0	Dicaffeoylquinic acid (3,4-DiCQA)
9	19.03	242.6, 327.0	516	515.5	354.1, 191.2	Dicaffeoylquinic acid (3,5-DiCQA)
10	20.04	327	342	341.3	179.1, 161.1	Caffeoyl glucopyranose
11	20.43	243, 329.4	516	515.5	354.1, 191.1, 179.1, 173.1, 135.1	Dicaffeoylquinic acid (1,5-DiCQA)
12	22.97	253.3, 349.7	478	477.4	315.3, 300.1, 270.9, 180.2	Isorhamnetin glucoside
13	26.57	327.0	516	515.5	191.1, 179.1, 173.1, 135.0	Dicaffeoylquinic acid (4,5-DiCQA)
14	30.20	263.9, 341.3	462	461.4	315.2, 284.8, 161.0, 132.7, 85.1	Kaempferol glucuronide
15	30.58	263.0, 333.0	448	447.4	285.4, 190.8, 153.1, 96.9	Kaempferol-3-o-glucoside

**Table 2:** Concentration of total phenolics and phenolic compounds in *H. tuberosus* leaves (cv. NanYu).

Phenolic compounds	Concentration^a^ (mg/g dry weight)
Caffeoylquinic acid (peak 2)^b^	0.063 ± 0.008^d^
3-o-Caffeoylquinic acid	7.752 ± 2.872^b^
Caffeoylquinic acid (peak 4)^b^	0.538 ± 0.081^d^
Caffeic acid	0.098 ± 0.052^d^
*p*-Coumaroyl-quinic acid	0.153 ± 0.061^d^
Feruloylquinic acid	0.527 ± 0.199^d^
3,4-Dicaffeoylquinic acid	0.618 ± 0.215^d^
3,5-Dicaffeoylquinic acid	4.900 ± 1.492^c^
Caffeoyl glucopyranose^c^	0.001 ± 0.319^d^
1,5-Dicaffeoylquinic acid	1.733 ± 0.567^d^
Isorhamnetin glucoside^d^	0.348 ± 0.057^d^
4,5-Dicaffeoylquinic acid	5.633 ± 2.990^bc^
Kaempferol glucuronide^d^	0.186 ± 0.034^d^
Kaempferol-3-o-glucoside^d^	1.020 ± 0.379^d^
Total phenolics^e^	23.570
Total phenolics^f^	30.159 ± 4.410^a^

^a^Values are expressed as mean ± SD of triplicate measurements; the means in a column followed by the same letters represent values that are not significantly different according to Duncan's test (*P* ≤ 0.05); ^b^quantified as 3-o-caffeoylquinic acid; ^c^quantified as caffeic acid; ^d^quantified as glucoside; ^e^sum of the individual phenolic compounds; and ^f^quantified as gallic acid equivalents.
